# Roles of programmed death protein 1/programmed death-ligand 1 in secondary brain injury after intracerebral hemorrhage in rats: selective modulation of microglia polarization to anti-inflammatory phenotype

**DOI:** 10.1186/s12974-017-0790-0

**Published:** 2017-02-14

**Authors:** Jie Wu, Liang Sun, Haiying Li, Haitao Shen, Weiwei Zhai, Zhengquan Yu, Gang Chen

**Affiliations:** Department of Neurosurgery & Brain and Nerve Research Laboratory, The First Affiliated Hospital of Soochow University, 188 Shizi Street, Suzhou, Jiangsu Province 215006 China

**Keywords:** Intracerebral hemorrhage, Secondary brain injury, Programmed death protein 1, Programmed death-ligand 1, Microglia polarization

## Abstract

**Background:**

Microglia and its polarization play critical roles in intracerebral hemorrhage-induced secondary brain injury. Programmed death protein 1/programmed death-ligand 1 has been reported to regulate neuroimmune cell functions. Signal transducers and activators of transcription 1 participate in microglia polarization, and programmed death protein 1/programmed death-ligand 1 could regulate the activation of signal transducers and activators of transcription 1. We herein show the critical role of programmed death protein 1/programmed death-ligand 1 in the polarization of microglia during intracerebral hemorrhage-induced secondary brain injury in rat models.

**Methods:**

An autologous blood intracerebral hemorrhage model was established in Sprague Dawley rats (weighing 250–300 g), and primary cultured microglia was exposed to oxyhemoglobin to mimic intracerebral hemorrhage in vitro. Specific siRNAs and pDNA for programmed death protein 1 and programmed death-ligand 1 were exploited both in vivo and in vitro.

**Results:**

In the brain tissue around hematoma, the protein levels of programmed death protein 1 and programmed death-ligand 1 and the interaction between them, as well as the phosphorylation of signal transducers and activators of transcription 1, were higher than that of the sham group and collectively peaked at 24 h after intracerebral hemorrhage. Overexpression of programmed death protein 1 and programmed death-ligand 1 ameliorated intracerebral hemorrhage-induced secondary brain injury, including brain cell death, neuronal degeneration, and inflammation, while their knockdown induced an opposite effect. In addition, overexpression of programmed death protein 1 and programmed death-ligand 1 selectively promoted microglia polarization to anti-inflammation phenotype after intracerebral hemorrhage and inhibited the phosphorylation of signal transducers and activators of transcription 1, suggesting that intracerebral hemorrhage-induced increases in programmed death protein 1 and programmed death-ligand 1 maybe a self-help.

**Conclusions:**

Enhancing the expressions of programmed death protein 1 and programmed death-ligand 1 may induce a selective modulation of microglia polarization to anti-inflammation phenotype for intracerebral hemorrhage treatment.

## Background

Intracerebral hemorrhage (ICH) accounts for ~15% of stroke cases in developed countries and ~50% or more in developing countries, especially in regions of Asia with high incidence of hypertension [1–3]. And ICH severely reduces the quality of life of patients, with a 5-year survival rate of ~30% [4, 5]. Beside blood clot-induced primary injury in the early phase [6], a series of chemical and immune responses known as secondary brain injury (SBI) seriously affect the prognosis of patients and is likely to be subjected to intervention [7–9].

Recent reports have shown that microglia/macrophages are the main regulatory cells in the immune defense response of the central nervous system (CNS) that affect subsequent inflammatory processes [10, 11]. After brain injury, a large number of microglia and macrophages around the hematoma rapidly respond and release effector molecules [12, 13]. Numerous recent studies have shown that in response to brain injury, different phenotypes of microglia/macrophages are observed and play various roles based on signals released in the microenvironment [11, 13]. Two main phenotypes of microglia/macrophages are pro-inflammatory phenotype and anti-inflammatory phenotype. Pro-inflammatory phenotype promotes inflammation and kills microorganisms, while anti-inflammatory inhibits inflammation and is involved in tissue repair and reconstruction [13]. Microglia activation and polarization during experimental intracerebral hemorrhage has been reported [14, 15].

Programmed death-1 (PD-1; also known as CD279 or PDCD1), which is a transmembrane glycoprotein of the CD28/cytotoxic T lymphocytes associated antigen-4 (CTLA-4) immunoglobulin superfamily, was first discovered in 1992 [16]. Its ligands, programmed death-ligand 1 (PD-L1) (B7-H1) and PD-L2 (B7-CD), are type I transmembrane proteins of the B7 family. PD-1 inhibits phosphatidylinositol-3-kinase (PI3K) and protein kinase B (Akt) activities [17]. The PI3K-Akt signaling pathway is involved in mediating phosphorylation of signal transducers and activators of transcription 1 (STAT1) [18], which has been reported to promote pro-inflammatory phenotype polarization of microglia [19]. PD-L2, another ligand of PD-1, was reported to have a threefold higher affinity for PD-1 compared with PD-L1 [20]. However, studies of the PD-1/PD-L2 signaling pathway have been rarely reported. Numerous studies have shown that PD-1/PD-Ls play important roles in mediating CNS disorders such as ischemic stroke [21], multiple sclerosis [22], and Alzheimer’s disease [23].

In conclusion, accumulating researches suggested a therapeutic potential of PD-1/PD-Ls in ICH-induced SBI via regulating microglia polarization. However, until now, no study has investigated the contribution of PD-1/PD-Ls to microglia polarization. Therefore, the aim of this study was to investigate the role of PD-1/PD-Ls and to assess the therapeutic potential of PD-1/PD-Ls in ICH-induced SBI.

## Methods

### Experimental animals

Adult male Sprague Dawley rats weighing 250–300 g were purchased from the Laboratory Animal Center, Medical College of Soochow University, Suzhou, Jiangsu, China. The animal experimental protocols were approved by the Animal Care and Use Committee of Soochow University and complied with the ARRIVE guidelines. All animals were housed in a quiet environment maintained at 18–22 °C with stable humidity, and animals had free access to food and water.

### ICH modeling in rats

**Fig. 1:**
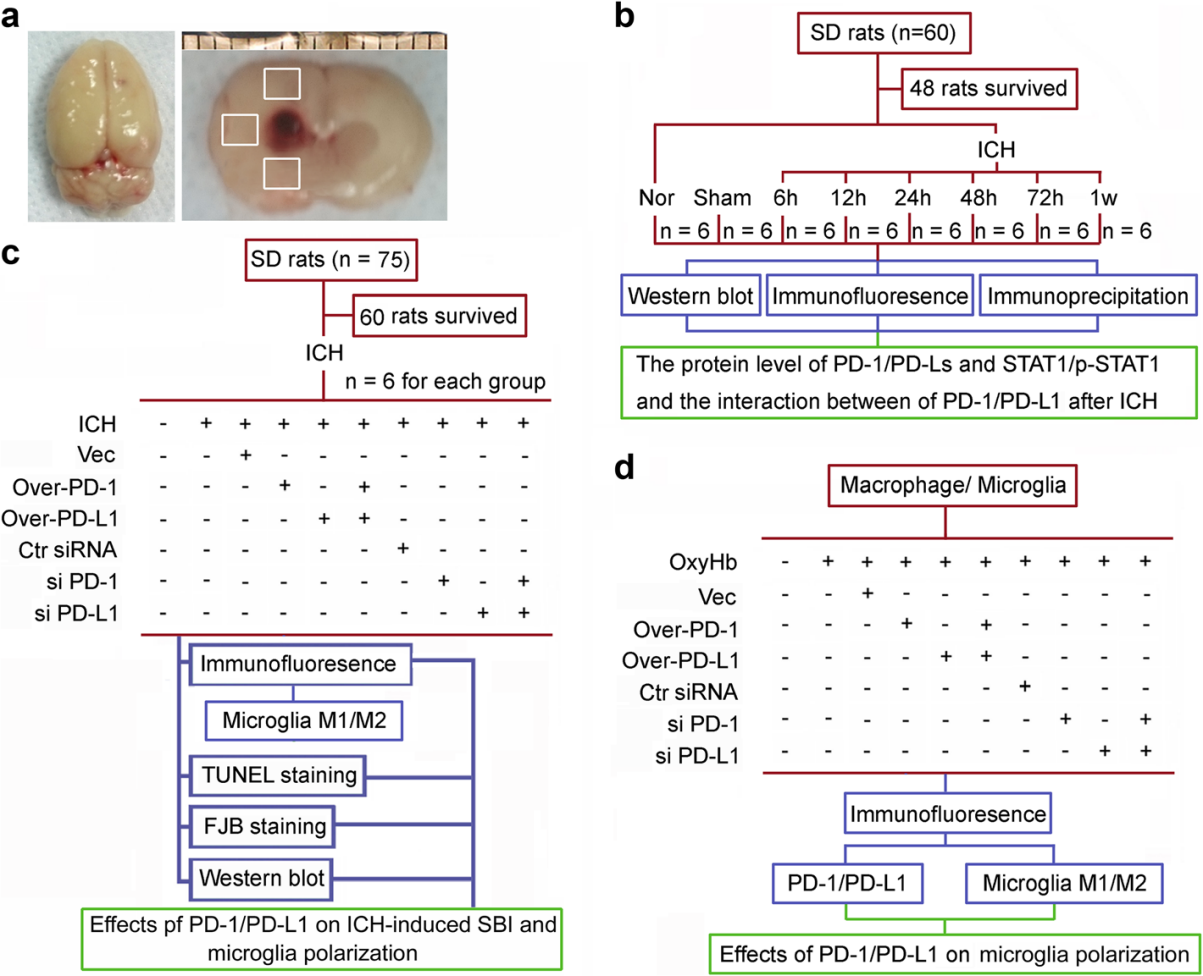
Flow chart showing rat brain tissue after ICH modeling and experimental design. **a** *Left panel*: representative image of completed isolation of rat brain tissue after cardiac perfusion. *Right panel*: brain section from a rat after ICH modeling. The *boxed areas* represent the brain regions used for western blot analysis and immunofluorescence staining. **b**, **c** Experimental design for in vivo experiment. **d** Experimental design for in vitro experiment

As described by Deinsberger et al., ICH modeling in rats was performed by injecting autologous arterial blood into the basal ganglia [24]. Rats were anesthetized by intraperitoneal injection of 10% chloral hydrate (36 mg/100 g bodyweight). After successful anesthesia, rats were placed in the prone position, and their heads were fixed with a stereotaxic frame (ZH-Lanxing B-type stereotaxic frame, Anhui Zhenghua Biological Equipment Co., Ltd. Anhui, China). The drill site of each animal was determined according to the stereotaxic atlas of the rat brain (arterial blood injection in this experiment was unified in the right basal ganglia) and was located 0.2 mm rostral to and 3.5 mm lateral to the anterior fontanelle (bregma). After stereotaxic positing, a cranial drill was used to drill a ~1-mm-diameter hole through the skull to the dura mater. A 100 μL microsyringe (Hamilton Company, Reno, NV) was fixed to a vertical position on the stereotaxic frame, and the needle head and dental drill were then aligned. The position of the stereotactic frame was fixed, and the microsyringe was removed. The hind limbs of the rat were then repositioned to face upward. The abdominal skin was disinfected, and a ventral midline laparotomy incision of ~2–3 mm in length was made. The caudal artery was carefully separated and exposed under a dissecting microscope. The region was rinsed with normal saline, and the caudal artery was punctured with a microsyringe needle to collect 100 μL of arterial blood without anticoagulant. After repositioning the animal in the stereotaxic frame, the microsyringe needle was slowly and vertically inserted through the dental drill until it reached a depth of ~5.5 mm. Next, 100 μL of autologous arterial blood was injected into the basal ganglia at a rate of 20 μL/min. After the injection, the needle was retained for 10 min to prevent bleeding. The microsyringe needle was slowly withdrawn, and sterilized bone wax was used to seal the hole in the skull. After confirming that there was no hemorrhage, the incision and head skin were sutured, and the tail incision was bandaged with pressure. Each animal received a subcutaneous injection of 5 ml normal saline for postoperative fluid replacement to prevent excessive loss of body fluid. Heart rate, blood pressure, and real-time body and rectal temperature were monitored for approximately 2 h, and temperature was maintained at 37.5 °C, until recovery from anesthesia. According to the four-level evaluation of neurological symptoms described by Bederson et al. [25], rats with neurological symptoms up to one to three level(s) were considered successfully established ICH models. Brain tissues were sampled 1 mm away from the hematoma to avoid potential red blood cell contamination for further study (Fig. 1a).

### Intracerebroventricular injection of pDNA or siRNA

The drilling site of the intracerebroventricular region for each rat was determined as described in previous studies [26, 27]. The relevant dosage of pDNA or small interfering RNA (siRNA) for intracerebroventricular injection was in accordance with manufacturer instructions. The drilling site was located 0.2 mm caudal to and 1.6 mm lateral to the anterior fontanelle (bregma). The depth of the drilling site was 4.5 mm.

### Microglia culture

Primary microglia-enriched cultures were prepared from the whole brains of 1-day-old pups as described previously [28]. Rats were fixed on a dissection platform and disinfected using 75% alcohol. The skin and skull were opened, and brain tissues were collected. Brain tissues were washed once in PBS and isolated from the brain stem. The meninges and blood vessels were removed, and the brain tissues were placed in serum-free DMEM/F12 culture medium. The tissues were cut into small pieces and transferred to centrifuge tubes. Next, 0.125% trypsin was added, and the brain tissue was digested in a water bath at 37 °C for 15 min. Trypsinization was terminated by adding a serum solution. After the tissue was precipitated, the supernatant was collected, and the remaining tissue was homogenized and trypsinized before filtering the tissue lysate. The filtered solution was centrifuged at 1500 rpm for 5 min. The supernatant was removed, and the cells were resuspended in complete culture medium before cell counting. The culture medium was changed every 2 days, and stratified cell layers were observed ~10 days later. Cells in the upper layer, which were mainly microglia, and a small number of oligodendrocytes, were semi-adherent and round with good light transmission. Cells in the lower layer were mainly astrocytes and neurons. Subsequently, culture flasks covered with glial cells/neurons were placed on a shaker and mixed at 180 rpm for 15 min. The culture medium was then collected, non-adherent cells were washed once with PBS, and the cells were centrifuged at 1500 rpm for 5 min. After removing the supernatant, cell pellets were resuspended with complete culture medium and incubated with 5% CO_2_ at 37 °C for 15–30 min. After changing the culture medium, the adhered cells in the culture flasks were purified microglia.

### Experimental groups

To examine PD-1 and PD-Ls expression at different time points after ICH, rats were divided into three groups: normal control group (*n* = 6), sham group (*n* = 6), and ICH group (*n* = 36) (Fig. 1b). Normal control group animals were not subjected to a procedure. Sham group animals underwent withdrawal of tail artery blood and sphenotresia via microsyringe needle puncture reaching basal ganglia but not blood vessels. ICH group animals were subjected to successful ICH modeling. The ICH group was randomly subdivided into six groups (*n* = 6 each) to collect brain samples at the indicated time points: post-ICH 6 h, post-ICH 12 h, post-ICH 24 h, post-ICH 48 h, post-ICH 72 h, and post-ICH 7 d. Rat brain tissues in the sham group were collected 48 h after sphenotresia.

To assess the effect of PD-1/PD-L1 on brain injury, rats were grouped as follows 24 h after ICH modeling (Fig. 1c). Seventy-five rats were randomly divided into ten groups: sham group (*n* = 6); ICH group (*n* = 6); vector group (*n* = 6/7, number of successful ICH models/total number of ICH models, same below); ICH + PD-1 overexpression group (*n* = 6/7); ICH + PD-L1 overexpression group (*n* = 6/9), ICH + PD-1 overexpression + PD-L1 overexpression group (*n* = 6/7); ICH + control siRNA group (*n* = 6/7); ICH + PD-1 siRNA group (*n* = 6/8); ICH + PD-L1 siRNA group (*n* = 6/8); and ICH + PD-1 siRNA + PD-L1 siRNA (*n* = 6/10). pDNA or siRNA was intracerebroventricularly injected into the rats 12 h before modeling. Animals were killed to collect brain tissue 24 h after ICH onsets. The serum was collected for enzyme-linked immunosorbent assay (ELISA). DNA and siRNA transfection efficiencies of each sample were verified using western blot analysis. Subsequently, effects of PD-1/PD-L1 on SBI and microglia polarization after ICH were determined using terminal dexynucleotidyl transferase(TdT)-mediated dUTP nick end labeling (TUNEL) staining, Fluoro-Jade B (FJB) staining, western blot analysis, and immunofluorescence staining.

To examine the effect of PD-1/PD-L1 on microglia polarization in vitro (Fig. 1d), cultured microglia were randomly divided into ten groups: control group, OxyHb group, OxyHb + vector group, OxyHb + PD-1 overexpression group, OxyHb + PD-L1 overexpression group, OxyHb + PD-1 overexpression + PD-L1 overexpression group, OxyHb + control siRNA group, OxyHb + PD-1 siRNA group, OxyHb + PD-L1 siRNA group, and OxyHb + PD-1 siRNA + PD-L1 siRNA group. pDNA or siRNA was transfected to the cultured microglia populations, and after 24 h, the cells were fixed with 4% formaldehyde. pDNA and siRNA transfection efficiencies of each sample were verified using western blot analysis, followed by microglia polarization analysis.

### pDNA and siRNA for PD-1/PD-L1 and transfection

Specific pDNA and siRNA for PD-1 and PD-L1 were obtained from Guangzhou Ribo Biotechnology Co., Ltd. (Guangzhou, China). To improve the knockdown efficiency, the interference efficiency of three different siRNAs (shown below) was test, and the most efficient one (I for PD-1, III for PD-L1) was used in the following study.

PD-1 siRNA sequences:

(I) Sense: 5′ CCACCUUCACCUGCAGUUU dTdT 3′
  Antisense: 3′ dTdT GGUGGAAGUGGACGUCAAA 5′
(II) Sense: 5′ CCGCUUCCAGAUCGUACAA dTdT 3′
  Antisense: 3′ dTdT GGCGAAGGUCUAGCAUGUU 5′
(III) Sense: 5′ CCUUCUGCUCAACAGGUAU dTdT 3′
  Antisense: 3′ dTdT GGAAGACGAGUUGUCCAUA 5′

PD-L1 siRNA sequences:

(I) Sense: 5′ GCAGAUUCCCAGUAGAACA dTdT 3′
  Antisense: 3′ dTdT CGUCUAAGGGUCAUCUUGU 5′
(II) Sense: 5′ GCCGAAGUGAUCUGGACAA dTdT 3′
  Antisense: 3′ dTdT CGGCUUCACUAGACCUGUU 5′
(III) Sense: 5′ GGUCAACGCAACAGCUAAU dTdT 3′
  Antisense: 3′ dTdT CCAGUUGCGUUGUCGAUUA 5′

pDNA and siRNA sequences were dissolved in RNase-free water to a concentration of 500 pmol/10 μL and then diluted with the same volume of transfection reagent. For each rat, 4 g of nucleic acid was injected intracerebroventricularly.

### TUNEL staining

Paraffin-embedded brain sections (4–6 μm thick) were incubated at 70 °C for 1 h, followed by dewaxing and rehydrating in xylene and graded ethanol (100, 95, 90, 80, and 70%) solutions. TUNEL fluorescence staining reagents (Roche) were used in accordance with the manufacturer’s instructions. Anti-quenching mounting medium was used to seal tissue sections between glass slides and cover slips. Brain sections were observed under a fluorescence microscope, and ImageJ software was used to analyze TUNEL staining. Six microscopic fields in each tissue section and three sections per rat were examined and photographed in parallel for TUNEL-positive cell counting. Microscopy was performed by an experienced pathologist blind to the experimental condition. The number of TUNEL-positive cells was assessed in ≥300 cells.

### FJB staining

After dewaxing the paraffin-embedded brain sections (described above), the samples were placed in the dark at room temperature and incubated with 0.06% KMnO_4_ solution for 15 min. Sections were then washed three times with PBS (5 min/wash), incubated with FJB working solution (containing 0.1% acetic acid solvent) for 60 min., and washed three times with PBS (5 min/wash). Brain sections on glass slides were air-dried at room temperature in a dark room and sealed with anti-quenching mounting medium and cover slips. Fluorescence microscopy was used for observation and photography. Six microscopic fields in each tissue section and three sections per rat were examined and photographed in parallel for FJB-positive cell counting. Microscopy was performed by an experienced pathologist blind to the experimental condition. The number of FJB-positive cells was assessed in ≥300 cells.

### Antibodies

Mouse anti-PD-1 antibody (MA5-15780), rabbit anti-PD-L1 antibody (PA5-20343), and rabbit anti-PD-L2 antibody (PA5-20344) were form Thermo Fisher (USA). p-STAT1 antibody (ab29045), STAT1 antibody (ab31369), rabbit anti-CD16 antibody (ab109223), anti-iNOS antibody (ab15323), anti-TNF alpha antibody (ab6671), anti-IL1 beta antibody (ab9722), anti-IL4 antibody (ab9811), and anti-IL10 antibody (ab9969) were from Abcam (Cambridge, MA, USA). Arginase 1 antibody (GTX109242) was form GeneTex (USA). Mouse anti-CD11b antibody (sc-516102), goat anti-CD206 antibody (sc-34577), mouse anti-β-actin (C4) antibody (sc-47778), normal mouse IgG (sc-2025), normal rabbit IgG (sc-2027), and normal goat IgG (sc-3887) were from Santa Cruz Biotechnology. Secondary antibodies for western blot analysis, including goat anti-rabbit IgG-HRP (sc-2004), goat anti-mouse IgG-HRP (sc-2005), and rabbit anti-goat IgG-HRP (sc-2922) were from Santa Cruz Biotechnology. Secondary antibodies for immunofluorescence microscopy, including Alexa Fluor-555 donkey anti-rabbit IgG antibody (A31572), Alexa Fluor-488 donkey anti-rabbit IgG antibody (A21206), Alexa Fluor-555 donkey anti-mouse IgG antibody (A31570), Alexa Fluor-488 donkey anti-goat IgG antibody (A11055), Alexa Fluor-488 goat anti-mouse IgG antibody (A-11001), Alexa Fluor-555 donkey anti-goat IgG antibody (A-21432), and Alexa Fluor-647 goat anti-rabbit IgG antibody (A32733) were from Invitrogen.

### Immunohistochemical study

The brain samples were fixed in 4% paraformaldehyde, embedded in paraffin, cut into 4-μm sections, and examined by immunofluorescence staining. Then, the sections were stained with primary antibodies (all diluted 1:200; from Santa Cruz Biotechnology, Inc.) and appropriate secondary antibodies (1:500 dilution; Santa Cruz Biotechnology, Inc.) as described. Normal rabbit IgG was used as a negative control for immunofluorescence assay (data not shown). Finally, sections were observed by a fluorescence microscope (OLYMPUS BX50/BX-FLA/DP70; Olympus Co., Japan). For fluorescence intensity assay, the relative fluorescence intensity was analyzed by use of ImageJ program by subtracting background. For positive cell counting, six microscopic fields in each tissue section and three sections per rat were examined and photographed in parallel for positive cell counting. Microscopy was performed by an experienced pathologist blind to the experimental condition. The number of positive cells was assessed in ≥100 cells.

### Western blot analysis

The brain tissue around hematoma was thoroughly ground on ice. RIPA lysis buffer (Beyotime Biotechnology) was used to lyse the brain tissue for 30 min. The lysate was then transferred to a centrifuge tube and centrifuged at 12,000 rpm for 5 min at 4 °C. The supernatant from each sample was collected and stored at −20 °C. Total protein extracted from each sample was quantified using a BSA protein concentration assay kit (Beyotime Biotechnology). Fifty micrograms of total protein from each sample was separated using SDS-PAGE. After transferring the protein gel to a PVDF membrane, corresponding proteins were probed with different antibodies and signals were detected using an ECL kit. ImageJ software was used to analyze optical density of bands.

### ELISA

The concentrations of IL-1β and TNF-α in serum were determined by corresponding ELISA kits (R&D Systems Inc., USA). These assays were performed according to manufacturer’s instructions, and these data were expressed relative to standard curves prepared for them.

### Co-immunoprecipitation (Co-IP)

Protein A + G agarose for IP was washed twice with lysis buffer. Total protein (100 μg) from each sample was diluted in 500 μL lysis buffer. Approximately 1 μg of conventional IgG, which was identical to the IgG used for IP, was added with 25 μL fully resuspended protein A + G agarose and gently mixed on a rocker at 4 °C for 30 min. Samples were then centrifuged at 1000 × *g* for 5 min at 4 °C, and the supernatant was collected for subsequent IP and removal of nonspecific antigens. Next, protein solution was added with 2 μg of the corresponding primary antibody, and the mixture was incubated on a rocker at 4 °C for 1 h. After thorough binding between antigen and primary antibody, 50 μL of fully resuspended protein A + G agarose was added, and the reaction was gently mixed on a rocker at 4 °C overnight. The mixture was then centrifuged at 1000 × *g* for 5 min at 4 °C, and the supernatant was discarded. The beads were washed three times with lysis buffer, and 75 μL 4× SDS sample buffer was added. The sample was boiled at 100 °C for 5 min and then centrifuged at 1000 × *g* for 5 min at 4 °C. The supernatant was collected and placed in a new Eppendorf tube. Each prepared sample was used directly in western blot analysis.

### Data analysis

All data are expressed as mean ± SEM. One-way ANOVA for multiple comparisons and Student-Newman-Keuls post hoc test were performed for TUNEL and FJB staining, immunofluorescence staining, immunoprecipitation analysis, and western blot. *p* < 0.05 was considered statistically significant.

## Results

### ICH-induced increase in the expressions of PD-1 and PD-L1 and the interaction between them in the brain tissue around hematoma in rats

**Fig. 2:**
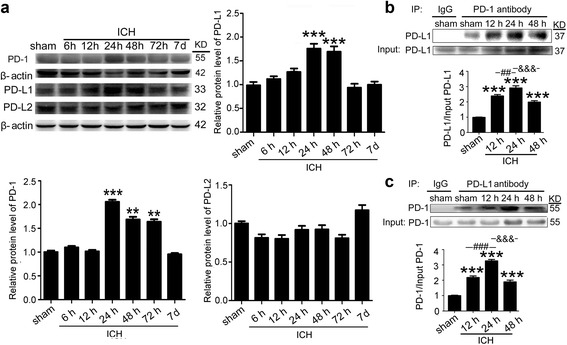
ICH increased the protein levels of PD-1/PD-Ls and the interaction between PD-1 and PD-L1. **a** Time course of the protein levels of PD-1, PD-L1, and PD-L2 in the brain tissue around hematoma after ICH. Representative western blot bands of PD-1, PD-L1, and PD-L2 and quantitative analysis of the relative protein level were shown. The mean value of sham group was normalized to 1.0. Data are expressed as mean ± SEM, *n* = 6. *Double asterisks* indicate *p* < 0.01, *triple asterisks* indicate *p* < 0.001 vs. sham group. **b**, **c** Immunoprecipitation analysis of the interaction between PD-1 and PD-L1 at indicated times after ICH. All values are means ± SEM, *n* = 6. *triple asterisks* indicate *p* < 0.001 vs. sham group, *triple pound signs* indicate *p* < 0.001

Expression of PD-1 protein in brain tissues from ICH model rats significantly increased at 24 h after ICH onsets. Results of western blot analysis showed that PD-1 protein expression in post-ICH 24 h, post-ICH 48 h, and post-ICH 72 h subgroups of ICH rats were significantly higher than that in the sham group (Fig. 2a). In addition, PD-L1 protein expression in post-ICH 24 h and post-ICH 48 h subgroups of ICH rats also increased. This enhanced expression of PD-L1 protein decreased in the post-ICH 72 h subgroup, which had similar PD-L1 protein expression levels as the control group (Fig. 2a). No significant changes in PD-L2 protein expression were found in the different groups (Fig. 2a). In addition, ICH also increased the interaction between PD-1 and PD-L1 at post-ICH 12 h, post-ICH 24 h, and post-ICH 48 h (Fig. 1b, c). And the protein levels of PD-1 and PD-L1 and the interaction between them, as well as the phosphorylation of STAT1, collectively peaked at 24 h after ICH. Therefore, we selected the post-ICH 24 h subgroup to study the effect of PD-1/PD-L1 on ICH-induced SBI.

### Effects of overexpression and knockdown of PD-1 and PD-L1 on brain cell death and neuronal degeneration in ICH rats

**Fig. 3:**
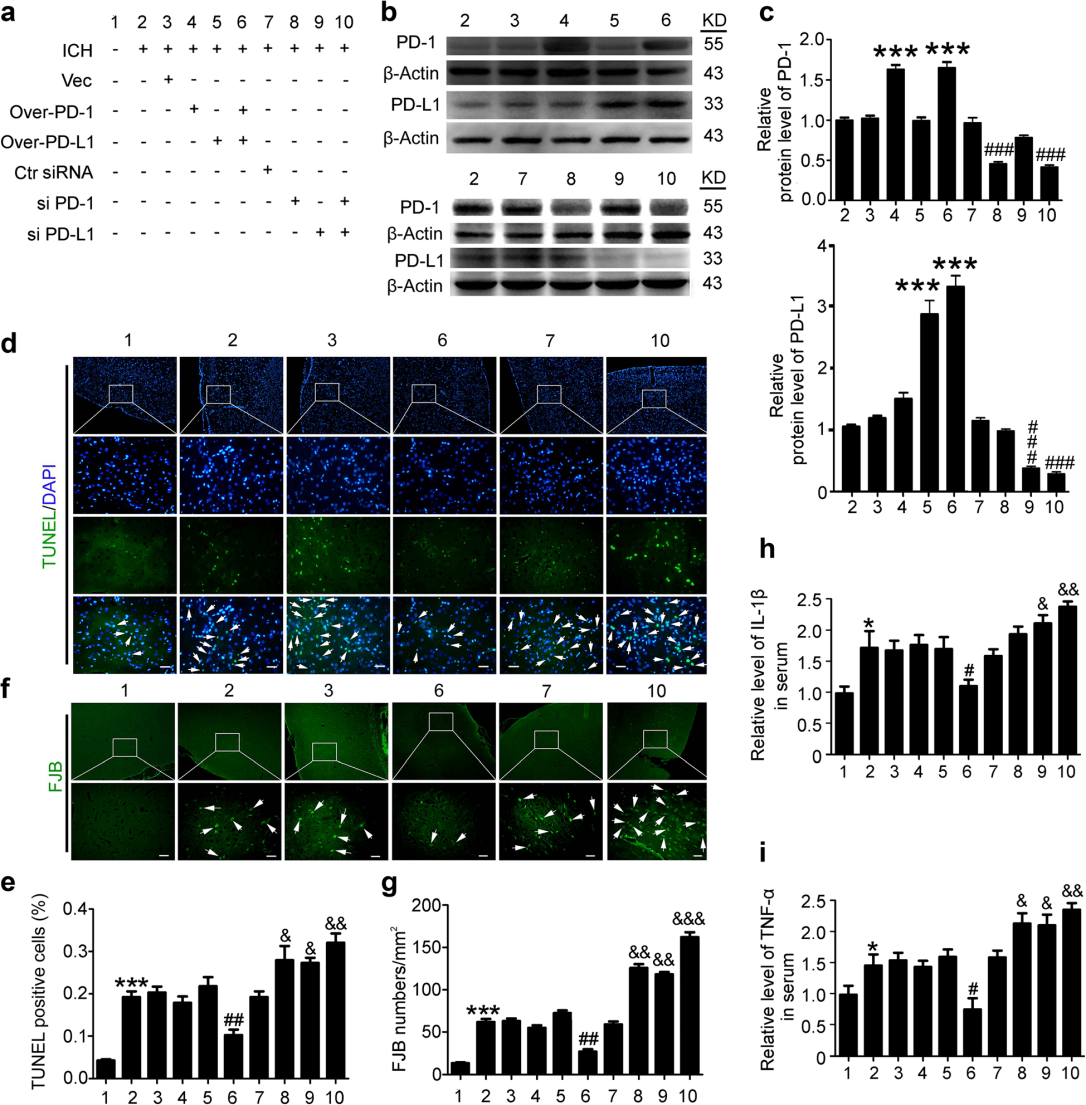
Effects of PD-1/PD-L1 overexpression and knockdown on brain cell death and neuronal degeneration after ICH. **a** ICH rats accepted intracerebroventricular injection of pDNA or siRNAs as indicated. **b** Western blot analysis of the efficiency of PD-1 and PD-L1 overexpression or knockdown in brain of ICH rats. Quantification of relative protein levels of PD-1 and PD-L1 was shown in **c. c** Data are expressed as mean ± SEM, *n* = 6. *Triple asterisks* indicate *p* < 0.001 vs. ICH + vector group; *triple pound signs* indicate *p* < 0.001 vs. ICH + control siRNA group. **d** Terminal deoxynucleotidyl transferase dUTP nick end labeling (TUNEL) staining. Sections were labeled by TUNEL (*green*) to detect apoptotic brain cells and counterstained with DAPI (*blue*) to detect nuclei. *Arrows* point to TUNEL-positive cells. *Scale bar* = 64 μm. **e** Percentage of TUNEL-positive cells. Data are expressed as mean ± SEM, *n* = 6. *Triple asterisks* indicate *p* < 0.001 vs. sham group; *double pound signs* indicate *p* < 0.01 vs. ICH + vector group; *ampersand* indicates *p* < 0.05, *double ampersands* indicate *p* < 0.01 vs. ICH + control siRNA group. **f** Fluoro-jade B (FJB) staining. *Arrows* point to FJB-positive cells. *Scale bar* = 64 μm. The number of FJB-positive brain cells was calculated. **g** Data are expressed as mean ± SEM, *n* = 6. *Triple asterisks* indicate *p* < 0.001 vs. sham group; *double pound signs* indicate *p* < 0.01 vs. ICH + vector group; *double ampersands* indicate *p* < 0.01, *triple ampersands* indicate *p* < 0.001 vs. ICH + control siRNA group. **h**, **i** ELISA assay of the contents of IL-1β and TNF-α in the serum. The mean values of the sham group were normalized to 1.0. Data are expressed as mean ± SEM, *n* = 6. *Single asterisk* indicates *p* < 0.05 vs. sham group; *Single pound sign* indicates *p* < 0.05 vs. ICH + vector group; *Single ampersand* indicates *p* < 0.05, *double ampersands* indicate *p* < 0.01 vs. ICH + control siRNA group

The experimental group of Fig. 3 is shown in Fig. 3a. Western blot assay showed that the protein levels of PD-1 and PD-L1 were significantly increased by pDNA transfection and decreased by siRNA transfection (Fig. 3b, c). TUNEL assay and FJB staining were used to assess the effects of PD-1/PD-L1 on brain cell death and neuronal degeneration in the brain after ICH modeling (Fig. 3d–g). The numbers of TUNEL- and FJB-positive cells in rat brains 24 h after ICH modeling were significantly higher than those in the sham group. However, overexpression of both PD-1 and PD-L1 significantly inhibited ICH-induced brain cell death and neuronal degeneration, while knockdown of them exerted an opposite effect. In addition, inflammation-associated molecules, including IL-1β and TNF-α, were tested. As shown in Fig. 3h, i, IL-1β and TNF-α were found to be significantly higher in the serum of the ICH group than that in the sham group. Compared with ICH group, the mean inflammatory cytokine contents were lower in overexpression group and higher in knockdown group. The results also showed that there were no significant differences between the vector group and PD-1 plasmid group or PD-L1 plasmid group, while there were significant differences between the control siRNA group and PD-1 siRNA group or PD-L1 siRNA group, suggesting that PD-1 and PD-L1complement each other in modulation of brain cell death and neuronal degeneration in ICH rats.

### Effects of PD-1 and PD-L1 on ICH-induced microglia polarization and STAT1 phosphorylation in rats

**Fig. 4:**
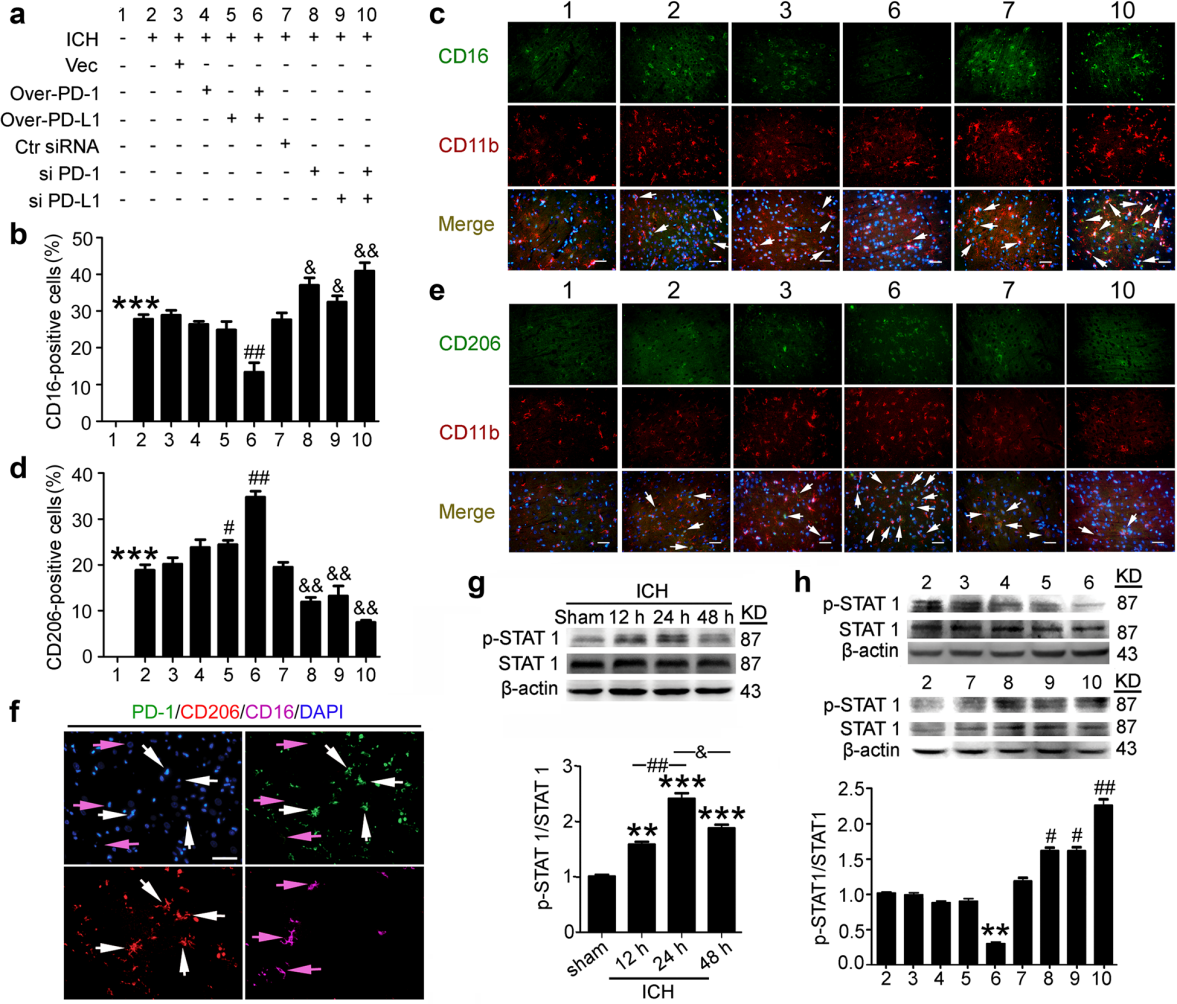
Effects of PD-1/PD-L1 overexpression and knockdown on ICH-induced microglia polarization and STAT1 phosphorylation. **a** ICH rats accepted intracerebroventricular injection of pDNA or siRNAs as indicated. Sections were stained for CD16/CD11b (pro-inflammatory microglia marker) or CD206/CD11b (anti-inflammatory microglia marker). Percentage of CD16-positive cells or CD206-positive cells was shown in **b** and **d** and representative images were shown in **c** and **e**. *Scale bar* = 64 μm. In **b** and **d**, data are expressed as mean ± SEM, *n* = 6. *Triple asterisks* indicate *p* < 0.001 vs. sham group; *single pound sign* indicates *p* < 0.05, *double pound signs* indicate *p* < 0.01 vs. ICH + vector group; *single ampersand* indicates *p* < 0.05, *double ampersands* indicate *p* < 0.01 vs. ICH + control siRNA group. **f** Sections of ICH + PD-1 overexpression + PD-L1 overexpression group were stained for PD1/CD16/CD206. *White arrows* point to CD206-positive cells with high fluorescence intensity of PD-1, and *purple arrows* point to CD16-positive cells with low fluorescence intensity of PD-1. *Scale bar* = 64 μm. **g** Western blot analysis and quantification of the phosphorylation level of STAT1. The mean values of the protein levels in the sham group were normalized to 1.0. Data are expressed as mean ± SEM, *n* = 6. *Double asterisks* indicate *p* < 0.01, *triple asterisks* indicate *p* < 0.001 vs. sham group; *double pound signs* indicate *p* < 0.01, *single ampersand* indicates *p* < 0.05. **h** Western blot analysis and quantification of the phosphorylation level of STAT1. The mean values of the protein levels in the ICH group were normalized to 1.0. Data are expressed as mean ± SEM, *n* = 6. *Double asterisks* indicate *p* < 0.01 vs. ICH + vector group; *single pound sign* indicates *p* < 0.05, *double pound signs* indicate *p* < 0.01 vs. ICH + control siRNA group

We next observed the effects of PD-1 and PD-L1 overexpression or knockdown on the microglia polarization in ICH rat brain (Fig. 4). The experimental group of Fig. 4 is shown in Fig. 4a. The results showed that ICH induced microglia polarization to a pro-inflammatory phenotype, as defined by CD16/CD11b-positive, and also to an anti-inflammatory phenotype, as defined by CD206/CD11b-positive (Fig. 4b–e). The results also showed that ICH mainly selectively modulated microglia polarizing to pro-inflammatory phenotype, which was ameliorated by overexpression of PD-1 and PD-L1 and aggravated by knockdown of PD-1 and PD-L1. To further elucidate the role of PD-1 and PD-L1 in microglia polarization, we co-stained brain sections of ICH + PD-1 overexpression + PD-L1 overexpression group for PD1, CD16 and CD11b or PD1, CD206, and CD11b (Fig. 4f). The results demonstrated that almost all of the CD206/CD11b-positive cells were co-labeled with high fluorescence intensity of PD1, while almost all of the CD16/CD11b-positive cells showed almost undetectable fluorescence intensity of PD1.

In addition, we used western blot analysis to measure the phosphorylation level of STAT1. Our results indicated that the phosphorylation level of p-STAT1 after ICH modeling was significantly higher than that in the sham group and peaked at 24 h after ICH onsets (Fig. 4g). And the phosphorylation level of STAT1 was decreased by overexpression of PD-1 and PD-L1 and further increased by knockdown of PD-1 and PD-L1 (Fig. 4h).

### Critical role of PD-1 and PD-L1 in microglia polarization in cultured microglia under OxyHb treatment

**Fig. 5:**
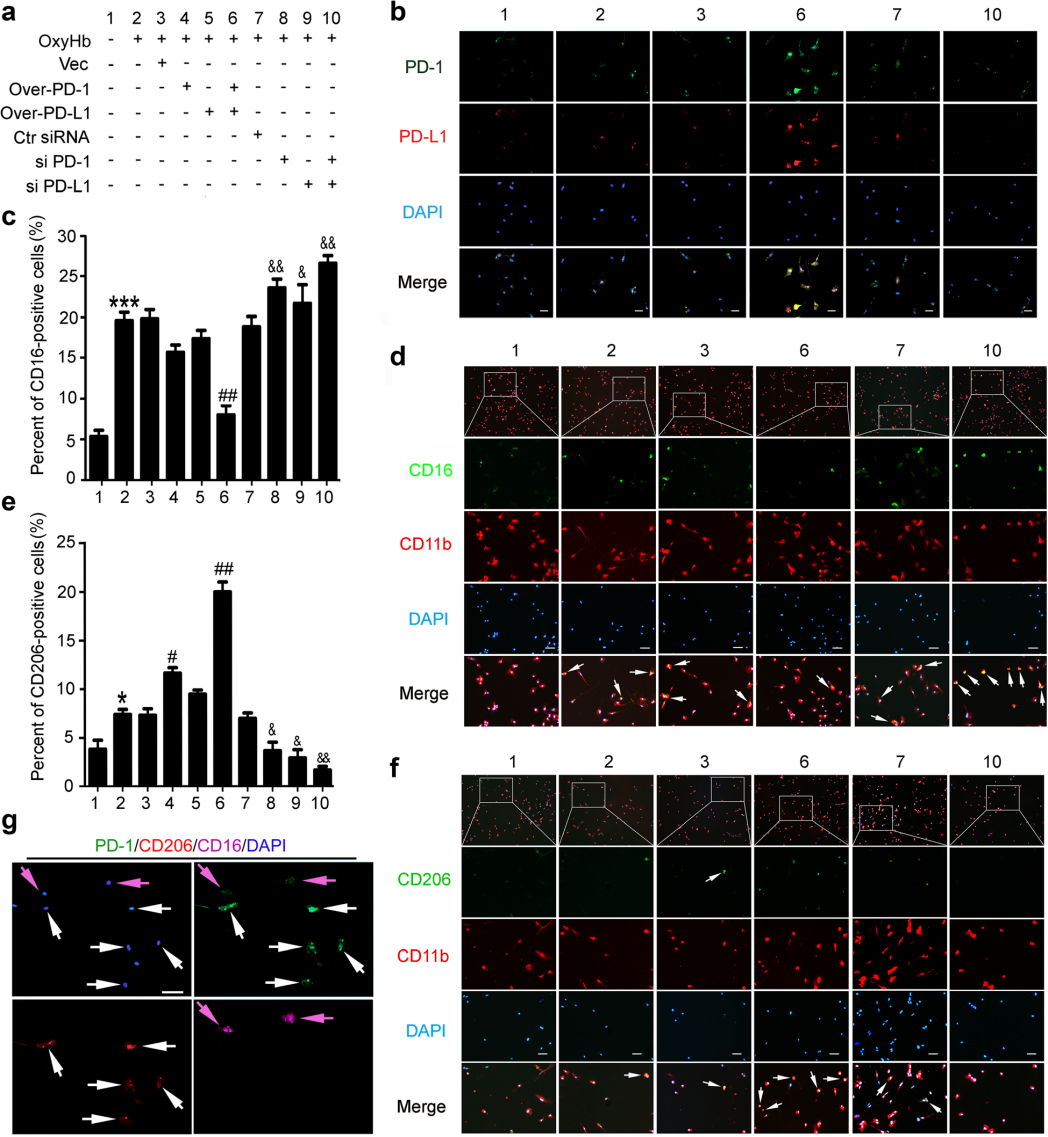
Effects of PD-1/PD-L1 overexpression and knockdown on the polarization of OxyHb-treated microglia. **a** Cultured microglia accepted transfection of pDNA or siRNAs as indicated. **b** The efficiency of pDNA and siRNA in cultured microglia was verified by immunofluorescence staining. *Scale bar* = 64 μm. Cultured microglia was stained for CD16/CD11b (pro-inflammatory microglia marker) or CD206/CD11b (anti-inflammatory microglia marker). Percentage of CD16-positive cells or CD206-positive cells was shown in **c** and **e** and representative images were shown in **d** and **f**. *Scale bar* = 64 μm. In **c** and **e**, data are expressed as mean ± SEM, *n* = 6. *Single asterisk* indicates *p* < 0.05, *triple asterisks* indicate *p* < 0.001 vs. sham group; *single pound sign* indicates *p* < 0.05, *double pound signs* indicate *p* < 0.01 vs. ICH + vector group; *single ampersand* indicates *p* < 0.05, *double ampersands* indicate *p* < 0.01 vs. ICH + control siRNA group. **g** Microglia of ICH + PD-1 overexpression + PD-L1 overexpression group was stained for PD1/CD16/CD206. *White arrows* point to CD206-positive cells with high fluorescence intensity of PD-1, and *purple arrows* point to CD16-positive cells with low fluorescence intensity of PD-1. *Scale bar* = 64 μm

We next observed the effects of PD-1 and PD-L1 overexpression or knockdown on microglia polarization in cultured microglia under OxyHb treatment (Fig. 5). The experimental group of Fig. 5 is shown in Fig. 5a. Firstly, the expression plasmid-mediated overexpression of PD1 and PD-L1 as well as the efficiency of siRNA-mediated knockdown in cultured microglia is verified by immunofluorescence staining (Fig. 5b). In addition, double-immunofluorescence analysis showed that OxyHb treatment induced microglia polarization both to a pro-inflammatory phenotype and an anti-inflammatory phenotype (Fig. 5c–f). The results also showed that OxyHb treatment mainly selectively modulated microglia polarizing to pro-inflammatory phenotype, which was ameliorated by overexpression of PD-1 and PD-L1 and aggravated by knockdown of PD-1 and PD-L1. To further elucidate the role of PD-1 and PD-L1 in microglia polarization, we co-stained brain sections of OxyHb + PD-1 overexpression + PD-L1 overexpression group for PD1, CD16 and CD11b or PD1, CD206, and CD11b (Fig. 5g). The results demonstrated that almost all of the CD206/CD11b-positive cells were co-labeled with high fluorescence intensity of PD1, while almost all of the CD16/CD11b-positive cells showed almost undetectable fluorescence intensity of PD1.

**Fig. 6:**
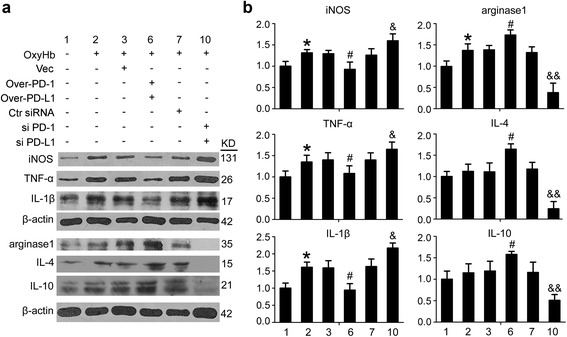
Changes in the expression of pro-inflammatory- and anti-inflammatory-like polarization markers in microglia under indicated treatment. **a** The immunoblots show TNF-α, IL-1β, iNOS, arginase1, IL-4, and IL-10 produced by microglia under indicated treatment. **b** The quantitative analyses of TNF-α, IL-1β, iNOS, arginase1, IL-4, and IL-10 in the immunoblots in **a**. Date = mean ± SEM, *n* = 6. *Single asterisk* indicates *p* < 0.05 vs. control group; *single pound sign* indicates *p* < 0.05 vs. ICH + vector group; *single ampersand* indicates *p* < 0.05, *double ampersands* indicate *p* < 0.01 vs. ICH + control siRNA group

In addition, as previously reported [29], the use of single phenotypic markers (CD206 and CD16) is not sufficient to identify microglia polarization. Inflammation-associated molecules, including TNF-α, IL-1β, iNOS, arginase1, IL-4, and IL-10, were tested to provide more information on the biological state of microglia after ICH and the roles of PD-1 and PD-L2 in the process (Fig. 6). The results showed that the mean protein levels of pro-inflammation molecules, including TNF-α, IL-1β, and iNOS, in cultured microglia were significantly increased by OxyHb treatment. However, among the anti-inflammation molecules, including arginase1, IL-4 and IL-10, only the protein level of arginase1 was increased after OxyHb treatment. These results suggested that OxyHb induced microglia polarization to a pro-inflammation phenotype. In addition, a significant decrease in the mean protein levels of TNF-α, IL-1β, and iNOS and a significant increase in the mean protein levels of arginase 1, IL-4, and IL-10 were observed in microglia with PD-1 and PD-L1 overexpression, while opposite trends were shown in microglia with PD-1 and PD-L1 knockdown, suggesting that PD-1 and PD-L1 selectively inhibited OxyHb-induced pro-inflammation polarization of microglia.

In addition, the mean protein levels of pro-inflammatory markers, including TNF-α, IL-1β, and iNOS, in cultured microglia were significantly increased by OxyHb treatment (Fig. 6). However, among the anti-inflammatory markers, including arginase 1, IL-4, and IL-10, only the protein level of arginase1 was increased after OxyHb treatment. These results suggested that OxyHb induced microglia activation and mainly promoted microglia polarization to pro-inflammatory phenotype. A significant decrease in the mean protein levels of pro-inflammatory markers and a significant increase in the mean protein levels of anti-inflammatory markers were observed in microglia with PD-1 and PD-L1 overexpression, while opposite trends were shown in microglia with PD-1 and PD-L1 knockdown, suggesting that PD-1 and PD-L1 selectively inhibited OxyHb-induced pro-inflammatory polarization of microglia.

## Discussion

**Fig. 7:**
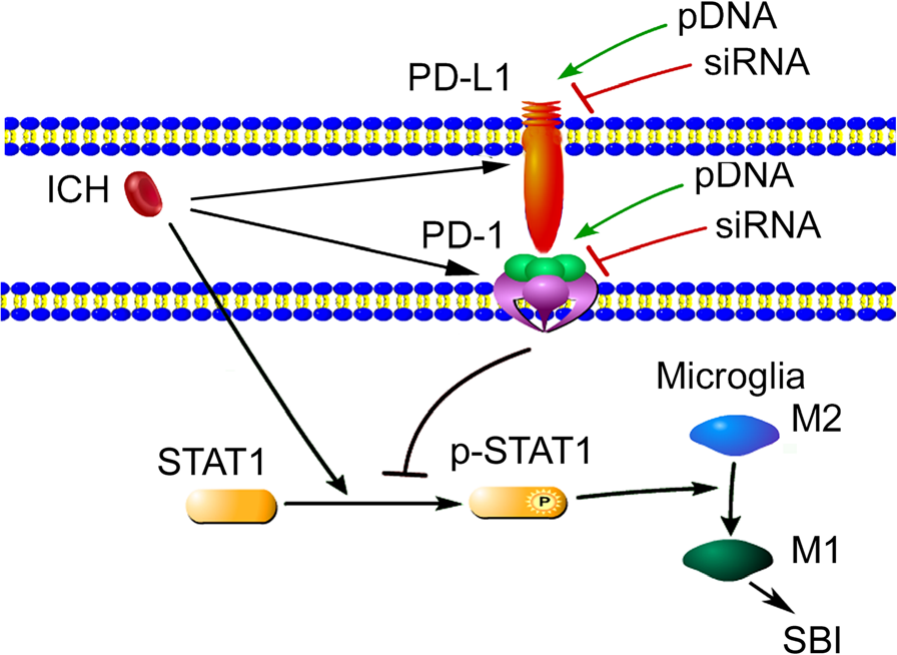
Possible mechanism of PD-1/PD-L1 in secondary brain injury after ICH modeling. After ICH, PD-1/PD-L1 protein expression in the brain tissue significantly increased. In addition, the proportion of STAT1 phosphorylation increased. Moreover, upregulation of PD-1/PD-L1 expression by overexpression suppressed the phosphorylation of p-STAT1, pro-inflammatory polarization of microglia, and subsequent SBI under ICH condition, while PD-1/PD-L1 knockdown induced an opposite effect

In this study, we established a rat model of ICH to determine whether PD-1/PD-Ls play a role in SBI and to elucidate the potential mechanisms. Our findings showed that PD-1/PD-L1 protein expression in brain tissue significantly increased after ICH. In addition, the proportion of STAT1 phosphorylation increased after ICH. These changes were more significant ~24 h after ICH. Overexpression of PD1 and PD-L1 inhibited STAT1 phosphorylation, which was an important factor for transforming microglia to the pro-inflammatory subtype. Thus, these results suggested that enhancing the expressions of PD-1 and PD-L1 may induce a selective modulation of microglia polarization to anti-inflammatory phenotype via STAT1 for ICH treatment (Fig. 7). This finding has not been reported in previous studies.

In a study of autoimmune encephalomyelitis, Carter et al. [30] showed that PD-1/PD-L1 but not PD-L2 are involved in T cell activation. In this study, we demonstrated that PD-1 and PD-L1 but not PD-L2 protein expression significantly reduced early brain injury (Fig. 2). Thus, we suggest that the PD-1/PD-L1 signaling pathway plays an important role in regulating the inflammatory response in nervous tissue. In the ICH rat model, PD-1 and PD-L1 protein expression and their binding to each other were elevated 24 h after ICH modeling, further indicating that PD-1/PD-L1 are involved in ICH-induced SBI. The severity of brain injury was alleviated increased protein expression of both PD-1 and PD-L1. In contrast, reduced PD-1 and PD-L1 protein expression increased the severity of brain injury, indicating a causal relationship between PD-1/PD-L1 and brain injury (Fig. 3).

Microglia/macrophages effectively regulate restoration and regeneration of the CNS. However, these molecules can act as double-edged swords, resulting in both destruction and restoration [31]. Further studies have demonstrated that activation of microglia/macrophages can convert the cells into two opposite phenotypes: pro-inflammatory microglia that release destructive inflammatory cytokines and anti-inflammatory microglia that release cytokines for protection and restoration [25, 32]. In addition, multiple members of the STAT family (i.e., STAT1, STAT3, and STAT6) are involved in morphological typing of microglia/macrophages. For example, STAT1 promotes transformation of microglia into pro-inflammatory microglia [19, 33], and STAT6 stimulates transformation of microglia into anti-inflammatory microglia [34]. Moreover, STAT1 and STAT6 have a mutually inhibitory relationship [35]. In this study, the ratio of p-STAT1/STAT1 was significantly increased following ICH (Fig. 4g), while ICH did not affect the level of p-STAT6/STAT6 significantly (data not shown). Among the different ICH rat model groups, the proportion of phosphorylated STAT1 was maximal 24 h after ICH. PD-1 reduced phosphorylation of STAT1, which was consistent with the result of a previous study [36]. The possible mechanism may be due to PD-1 blocking the PI3K-Akt pathway, which inhibits promotion of phosphorylation of STAT1 [18]. In a mouse model of spinal cord injury, inhibition of PD-1 promotes transformation of microglia/macrophages into pro-inflammatory microglia [36]. These findings support our hypothesis that PD-1/PD-L1 is involved in inhibiting pro-inflammatory polarization of microglia under conditions of ICH [36].

There are several limitations of this study. In vitro experiment showed OxyHb maybe an incentive for the increase in the expressions of PD-1 and PD-L1. However, due to lacking studies of other contents of hematoma, this study cannot draw the mechanism underlying ICH-induced increase in the expressions of PD-1 and PD-L1. In addition, whether ICH-induced increase in the expressions lead to ICH-induced increase in the interaction between PD-1 and PD-L1 is also not answered in this study. It also reported that the PD-L1-mediated inflammatory response expanded the infarct size in experimental stroke model [37]. The possible mechanism may involve binding between PD-L1 and CTLA-4. CTLA-4 inhibits Akt phosphorylation through activation of protein phosphatase 2 (PP2A) [17, 38]. And a study has shown that PD-1 can inhibit PD-L1 protein expression by promoting transcription of MicroRNA 513 [39] to negatively regulate PD-L1 and play a neuroprotective effect in nervous tissue. The effect of CTLA-4 and MicroRNA 513 on microglia polarization in ICH condition deserves further study.

## Conclusions

Microglia polarization is part of the basic machinery of brain injury. PD-1/PD-L1, a typical member of immunoglobulin superfamily, plays a critical role in neuroimmune cell functions. On the other hand, much attention has been also paid to the role of STATs in microglia polarization. In this study, using in vivo and in vitro models of relevance to ICH, we found a critical role of PD-1/PD-L1 through STAT1 in the polarization of microglia. These findings suggest PD-1/PD-L1 as promising pharmacological targets for the treatment of hemorrhagic brain injury.

## Abbreviations

Akt: Protein kinase B
CNS: Central nervous system
Co-IP: Co-immunoprecipitation
CTLA-4: Cytotoxic T lymphocytes associated antigen-4
FJB: Fluoro-Jade B
ICH: Intracerebral hemorrhage
PD-1: Programmed death protein 1
PD-L1: Programmed death-ligand 1
PI3K: Phosphatidylinositol-3-kinase
SBI: Secondary brain injury
STAT1: Signal transducers and activators of transcription 1
TUNEL: Terminal dexynucleotidyl transferase(TdT)-mediated dUTP nick end labeling
